# Revisiting the Complex Pathosystem of Huanglongbing: Deciphering the Role of Citrus Metabolites in Symptom Development

**DOI:** 10.3390/metabo10100409

**Published:** 2020-10-13

**Authors:** Yasser Nehela, Nabil Killiny

**Affiliations:** 1 Department of Plant Pathology, Citrus Research and Education Center, University of Florida, 700 Experiment Station Rd., Lake Alfred, FL 33850, USA; yasser.nehela@ufl.edu; 2 Department of Agricultural Botany, Faculty of Agriculture, Tanta University, Tanta 31511, Egypt

**Keywords:** huanglongbing, ‘*Candidatus* Liberibacter’, Asian citrus psyllid, *Diaphorina citri*, citrus, metabolites, phytohormones, polyamines, blotchy mottle, fruit drop, root damage, flushing cycles

## Abstract

Huanglongbing (HLB), formerly known as citrus greening disease, is one of the most devastating bacterial diseases in citrus worldwide. HLB is caused by ‘*Candidatus* Liberibacter asiaticus’ bacterium and transmitted by *Diaphorina citri.* Both ‘*Ca.* L. asiaticus’ and its vector manipulate the host metabolism to fulfill their nutritional needs and/or to neutralize the host defense responses. Herein, we discuss the history of HLB and the complexity of its pathosystem as well as the geographical distribution of its pathogens and vectors. Recently, our recognition of physiological events associated with ‘*Ca.* L. asiaticus’ infection and/or *D. citri*-infestation has greatly improved. However, the roles of citrus metabolites in the development of HLB symptoms are still unclear. We believe that symptom development of HLB disease is a complicated process and relies on a multilayered metabolic network which is mainly regulated by phytohormones. Citrus metabolites play vital roles in the development of HLB symptoms through the modulation of carbohydrate metabolism, phytohormone homeostasis, antioxidant pathways, or via the interaction with other metabolic pathways, particularly involving amino acids, leaf pigments, and polyamines. Understanding how ‘*Ca*. L. asiaticus’ and its vector, *D. citri*, affect the metabolic pathways of their host is critical for developing novel, sustainable strategies for HLB management.

## 1. Introduction

Vector-borne phytopathogens are responsible for more than 700 plant diseases and can cause high-impact economic losses worldwide [1,2,3]. Most, if not all, of these pathogens are vascular-limited colonizers. The plant vascular system includes phloem and xylem, where both satisfy the nutritional needs of many phytopathogens [4,5,6]. However, most of these pathogens colonize the phloem tissue specifically because of its richness in photosynthates, particularly sugars, amino acids, and other soluble organic compounds [4,7]. Moreover, several sap-sucking insects, such as aphids, psyllids, whiteflies, and leafhoppers, depend on the phloem contents for their feeding [6]. These hemipterans can transmit a wide range of pathogens such as viruses, bacteria, mollicutes, and fungi [8,9], even though few of them can transmit bacterial phytopathogens due to vector specificity, while the majority are vectors for viral diseases [6]. Vector-borne bacterial phytopathogens including *Xylella fastidiosa*, *Spiroplasma* spp., ‘*Ca*. Liberibacter spp.’, and ‘*Ca*. Phytoplasma spp.’ can cause several destructive diseases in both perennial and annual crops worldwide.

Although most of the Liberibacter species have not been cultured and Koch’s postulates have not yet been fulfilled, they have been reported as causal agents of huanglongbing (HLB), zebra chip (ZC), and several other plant diseases. HLB (aka citrus greening disease) is associated with a fastidious, phloem-limited, plant pathogenic bacterium ‘*Candidatus* Liberibacter spp.’ [10,11,12,13]. Based on the geographical distribution and characteristic 16S rDNA sequence, three Liberibacter species that were proposed to be associated with HLB include ‘*Ca*. L. africanus’, ‘*Ca*. L. americanus’, and ‘*Ca*. L. asiaticus’ [10,12,13,14,15,16,17,18,19,20,21]. ‘Ca. L. africanus’ is a heat-sensitive species originated in Africa, whereas ‘*Ca.* L. asiaticus’ is a heat-tolerant species originated in Asia and spread in the Arabian Peninsula, Africa, Asia, and the Americas. On the other hand, ‘*Ca*. L. americanus’ is also a heat-tolerant species but spread only in Brazil [12,13,14,18,20,21,22]. Among the three *Ca*. Liberibacter species, ‘*Ca.* L. asiaticus’ is the dominant species and is causing huge economic losses to citrus production worldwide [12,13]. 

The tree-to-tree transmission of ‘*Ca*. Liberibacter spp.’ can occur by graft inoculation of contaminated budwood; however, they are mainly transmitted by citrus psyllid vectors [23] and there is no evidence for their transmission by seeds. The African psyllid citrus, *Trioza erytreae* Del Guercio (Hemiptera: Triozidae), handles the transmission of ‘*Ca*. L. africanus’ in the Middle East, Mauritius, Reunion, and Africa [12,15], whereas the Asian citrus psyllid, *Diaphorina citri* Kuwayama (Hemiptera: Liviidae), handles the transmission of both ‘*Ca*. L. americanus’ in Brazil and ‘*Ca.* L. asiaticus’ in Asia and the Americas, and has recently spread to other citrus-growing regions [12,16,24]. Both psyllids in general and *D. citri* particularly may be the most serious phloem-sucking insect of citrus globally, especially when ‘*Ca.* L. asiaticus’ or ‘*Ca*. L. americanus’ also are present [23].

‘*Ca.* Liberibacter spp.’ can infect most, if not all, citrus cultivars, hybrids, and relatives. However, some citrus species are more tolerant to HLB than others [25,26,27,28,29]. For instance, HLB severely affects sweet orange, mandarin, and tangelo [26]; however, Mexican lime (*Citrus aurantiifolia* (Christm.) Swingle) was less susceptible even though it is a favored host for *D. citri* [30]. Additionally, several rutaceous genera other than *Citrus* sp. can harbor ‘*Ca.* Liberibacter spp.’ [17,21,22,23].

The symptoms of the three general forms of HLB (the Asian, African, and American form) are similar. The Asian form is more heat tolerant and expresses symptoms in both cool and warm conditions. The Asian form is more severe on most, if not all, commercial citrus species, causing dieback and can eventually result in tree death [12,31,32,33,34], whereas the African form of HLB expresses symptoms only in cool conditions (20–25 °C) and might be suppressed by long exposure to temperatures above 30 °C. The African form is more severe on mandarins and sweet orange, while lemons, limes, grapefruit, and pummelos are more tolerant [12,34,35,36]. The American form is presumptively closer to the Asian form in its symptom expression and severity; however, it appeared to be heat intolerant, similar to the African form [34]. Generally, HLB symptoms are more apparent during cooler seasons as compared with symptoms in warmer ones [37].

Recently, our knowledge about the molecular and physiological events associated with ‘*Ca*. L. asiaticus’ infection and/or *D. citri*-infestation has greatly improved. However, the mechanisms of HLB symptom development remain unexplored. Herein, we will discuss the potential roles of citrus metabolites in the development of the most characteristic symptoms of HLB. Understanding the relationships between different metabolic pathways and their roles in HLB symptom development may clarify the defense mechanisms of citrus against ‘*Ca*. L. asiaticus’ and *D. citri* toward finding novel, sustainable strategies for HLB management.

## 2. Symptoms of Huanglongbing

HLB-infected trees can show a wide range of symptoms that could resemble those of other disorders and nutrient deficiencies. This makes it very hard to diagnose the disease in its early stages. However, there are some unique characteristic symptoms of HLB disease. HLB-infected trees will usually develop yellowing on an individual limb or in one sector of a tree’s canopy, hence the name of the disease, which was described as the “yellow shoot disease” in English [38,39]. The other branches of the tree could remain healthy or symptomless, giving a sectored appearance, or symptoms may appear on different parts of the tree. The common symptoms of HLB will be described in the following paragraphs.

### 2.1. Symptoms of HLB on Leaves

**Figure 1:**
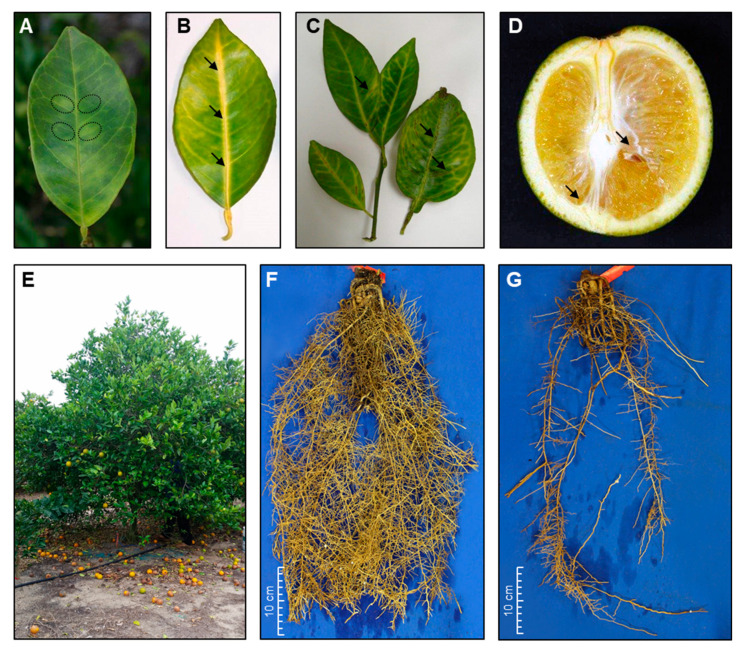
The characteristic symptoms of huanglongbing (HLB) disease. (**A**) Blotchy mottle symptom on HLB-positive citrus leaves with asymmetric patterns of discolorations around the leaf midvein; (**B**) Yellow vein symptom on HLB-positive citrus leaves; (**C**) Leathery leaves with enlarged corky veins from HLB-affected trees; (**D**) Lopsided, asymmetric, and small-sized fruits from HLB-affected trees; (**E**) HLB-induced preharvest fruit drop; (**F**,**G**) The root system of HLB-free (healthy) and HLB-positive trees, respectively. Photos used in panels (**A**–**D**) were provided by Dr. Pedro C. Gonzalez-Blanco whereas the photo used in panel (**E**) was provided by Dr. Tripti Vashisth, Department of Horticultural Sciences, Citrus Research and Education Center, University of Florida. Photos used in panels (**F**) and (**G**) were provided by Dr. Evan Johnson, Department of Plant Pathology, Citrus Research and Education Center, University of Florida.

The most characteristic foliar symptom of HLB is blotchy mottle [12,34] (Figure 1A). HLB-affected leaves can be normal-sized or small, developing a pattern of yellow and green areas lacking clear limits between the colors, giving a “blotchy mottle” appearance [40]. The chlorotic patterns are asymmetrical on the two halves of the citrus leaf [12] and resemble those induced by nutritional deficiencies such as zinc deficiency [22,37,40,41]. Nevertheless, HLB symptoms usually occur on a single branch and subsequently extend randomly in the tree canopy over several years, whereas zinc deficiency usually appears uniformly throughout the canopy. Moreover, the yellow coloration of HLB usually crosses the veins and is asymmetrically displayed on the leaf blade, whereas zinc deficiency usually shows a symmetrical yellowing throughout the leaf. Finally, blotchy mottle symptom is most frequently found on newly mature hardened leaves and fades with age. In addition to the blotchy mottle symptom, HLB-affected leaves may be thicker and leathery with enlarged corky yellow veins [12,34] (Figure 1B,C). At severe infection, leaves show what is called “rabbit ears” that are small, upright shoots with compressed internodes (the length of the internodes is shorter than usual), and this is might be followed by leaf drop and twig dieback.

### 2.2. Symptoms of HLB on Citrus Fruits

HLB-infected trees usually produce multiple off-season flowers (most of which fall prematurely), and small-sized, lopsided, and asymmetric fruits (Figure 1D). As the disease progresses, the fruit peel becomes thicker, and the fruit may ripen only partially, remaining green at the bottom with an inversion of colors, hence the other name of the disease, “citrus greening” [12,34]. The greening first starts at the stylar end, turning from green to yellow/orange in the peduncular end. Moreover, infected fruits may have a curved axis, contain smaller brownish aborted seeds, and have a tendency to drop prematurely. Premature HLB-induced fruit drop (Figure 1E) is a major economic symptom resulting in excessive yield reduction [40]. Furthermore, most of the HLB-symptomatic fruits do not make it to processing due to premature HLB-induced fruit drop or their elimination by sizing machines [37,42]. Nevertheless, fruits from heavily HLB-infected trees taste bitter and contain lower levels of soluble acids with reduced weight and juice content. Juice from HLB-infected fruit often has a lower sugar content, higher acid content, and more of the bitter flavonoids limonin and nomilin, which all contribute to bitter, off-flavor traits in juice. However, some of these fruits enter the processing stream because there are not unhealthy enough or have normal-sized fruits [37,42].

### 2.3. Symptoms of HLB on Roots

The root systems of the HLB-symptomatic trees are poorly developed and show significant decay of feeder and lateral roots and very few fibrous roots (Figure 1F,G), first thought to be due to nutrient starvation [22,43]. Johnson et al. [44], however, found that the loss of fibrous root mass was not due to phloem collapse or carbohydrate starvation but to an unidentified bacterial effector or toxin, i.e., as a direct result of the presence of the bacteria. In addition, HLB-infected trees usually have fewer roots, lower root dry weight, and lower root density compared to presumptively healthy and HLB-symptomless trees [44]. The roots, as the first phloem sink, were found to serve as a reservoir for ‘*Ca*. L. asiaticus’ and preceded foliar symptoms [44].

### 2.4. Symptoms of HLB on the Whole Tree

HLB-infected trees appear stunted and smaller compared with HLB-free trees. Due to the irregular distribution of ‘*Ca*. L. asiaticus’ within the infected trees, symptoms appear irregularly distributed on the tree canopy. As the disease progresses, severely infected trees may become sparsely foliated with the top third of the canopy being thin, and with small leaves that point upward. Eventually, HLB-infected trees develop extensive twig and limb dieback, may go into a complete decline, collapse, and ultimately die. Tree death occurs from several months to years after ‘*Ca*. L. asiaticus’ infection [12,22,40,43,45,46]. Additionally, HLB potentially enhances the tree’s susceptibility to other pests such as the citrus longhorned beetle, *Anoplophora chinensis* [23,43].

## 3. Deciphering the Role of Citrus Metabolites in HLB Symptom Development

Due to the lack of a sustainable cure for HLB and the relatively rapid progression of the disease in citrus groves, it became vital to investigate the molecular mechanisms behind the disease to better understand the pathogenesis of ‘*Ca*. L. asiaticus’ and how it uses these mechanisms to develop symptoms in its host [47,48]. Although several recent studies have provided insights into the role of leaf metabolites in the citrus response against ‘*Ca.* L. asiaticus’ [25,49,50,51,52,53,54,55,56,57,58,59,60,61,62,63,64], the role of these metabolites in symptom development is still poorly understood.

It has been suggested that three major molecular mechanisms might be associated with symptom development of HLB [65]. These mechanisms include the following: (I) disorder of carbohydrate metabolism associated with source–sink disruption due to starch accumulation in leaves, but not in fruits, possibly due to the upregulation of glucose-phosphate transport (GPT) [65]; (II) imbalance of stress-associated phytohormones of citrus plants, particularly jasmonic-salicylic acid crosstalk; and (III) activation of detoxification proteins, particularly glutathione-S-transferases (GSTs) and modulation of antioxidant pathways [65].

**Figure 2:**
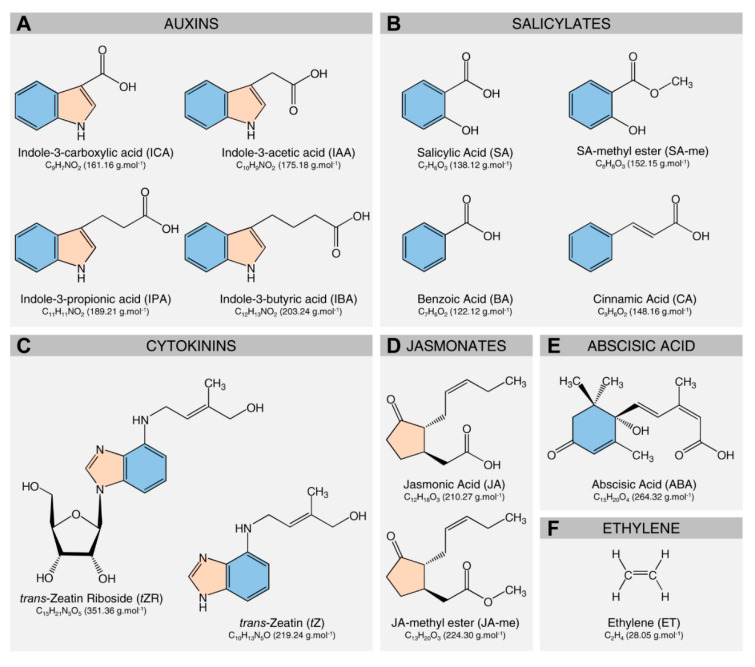
The chemical structures of different phytohormones discussed throughout this review. (**A**) auxins, (**B**) salicylates, (**C**) cytokinins, (**D**) jasmonates, (**E**) abscisic acid, and (**F**) ethylene. Molecular weights (g·mol^−1^) are mentioned between parentheses beside the chemical formula of each compound.

However, HLB symptom development is a complicated process, and probably reliant on a complex metabolic network consisting of at least these three mechanisms that are mainly regulated by phytohormones (Figure 2). In addition, previous studies demonstrated that ‘*Ca.* L. asiaticus’ infection induces significant changes in the profiles of both primary and secondary metabolites including amino acids [25,50,51,55,61,62], organic acids [50,55,61], fatty acids [25,55], phytohormones [58,59], and polyamines (PAs) [49,52,53,54,62]. However, the contribution of each group of metabolites to the symptom progression of HLB is poorly understood.

In the current review, we will discuss previous metabolomic and transcriptomic studies to better understand the roles of citrus metabolites in the symptom development of HLB disease. We believe that the metabolic responses of citrus plants may result from host cellular functions for defense reactions, or it may result from the manipulation of metabolic pathways by ‘*Ca.* L. asiaticus’ and/or *D. citri* for their benefit (to fulfill their nutritional needs).

### 3.1. The Role of Citrus Metabolites in the Development of Blotchy Mottle Symptom

The most characteristic symptom of HLB is blotchy mottle, which is an asymmetrical discoloration/chlorosis across the mid-vein of the leaf with patches of yellow and green islands [12,66]. Symptoms of HLB are induced by both the pathogen and its vector, and are due to alterations in many physiological aspects such as phytohormonal levels, carbohydrate status [58,59], and carotenoid content [56,67,68]. Previously, we demonstrated that infection with ‘*Ca.* L. asiaticus’ and infestation with its vector *D. citri* negatively affected the profile of citrus leaf pigments with a greater effect for ‘*Ca.* L. asiaticus’ [56].

**Figure 3:**
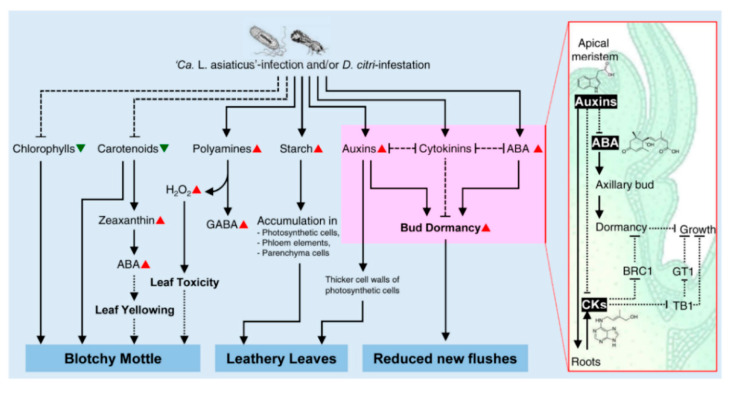
Hypothetical model of the potential roles of citrus metabolites in the development of huanglongbing (HLB) symptoms. Briefly, HLB symptoms are induced by both the pathogen and its vector due to alteration in many physiological aspects such as phytohormones, polyamines, carbohydrate status, and pigment content. The most characteristic symptom of HLB, blotchy mottle, might be due to the degradation of both chlorophylls and carotenoids. Additionally, ABA could induce leaf yellowing, which might help the development of blotchy mottle symptom. Furthermore, polyamines might be involved in HLB symptom development via the production of H_2_O_2_, which may eventually become toxic to the leaf tissue and cause the characteristic blotchy mottle symptom that appears after ‘*Ca*. L. asiaticus’ infection. Another characteristic symptom of HLB is the leathery leaves, which could be due to the extensive accumulation of starch grains in the photosynthetic cells, phloem elements, vascular parenchyma, and all other parenchyma cells of the HLB-symptomatic leaves. In addition, we suggest that auxin accumulation in HLB-infected leaves might be involved in the development of leathery leaf symptom via the formation of thicker cell wall in photosynthetic cells. Moreover, it is well-known that ‘*Ca*. L. asiaticus’ infection reduces the production of new flushes on infected trees. We believe that the reduced new flushes symptom could be due to the phytohormonal imbalance, particularly the auxin/cytokinin ratio. The solid lines with arrows signify positive reaction, the dashed lines with bar-ends indicate negative correlation, and round-dotted lines represent hypothetical mechanisms or uncharacterized elements, with arrows (positive) or bar-ends (negative). The red triangles signify increased levels, whereas green upside-down triangles signify decreased levels of these compounds upon the infection with ‘*Ca*. L. asiaticus’ and/or infestation with *D. citri*.

Briefly, out of 15 reported pigments, the infection with ‘*Ca.* L. asiaticus’ enhanced the accumulation of only zeaxanthin but diminished that of the other pigments (13 compounds) while infestation with *D. citri* boosted the chlorophyllide *a* content but reduced that of other pigments (nine pigments) [56]. Moreover, all chlorophyll biosynthetic genes, except chlorophyllase (*Chlase*s, also known as *CLH*s), were downregulated in ‘*Ca.* L. asiaticus’-infected plants [56]. Likewise, infection with ‘*Ca.* L. asiaticus’ decreased the expression levels of 15 genes involved in the early/shared steps of the carotenoid biosynthesis pathway, while it upregulated 14 genes involved in the late/specific steps [56]. In the carotenoid biosynthesis pathway, the early/shared steps are plastid-localized [69] and it has two characteristic branches; the alpha-arm refers to the alpha-carotene branch that leads to lutein biosynthesis and the beta-arm refers to the beta-carotene branch that directs to ABA biosynthesis. Interestingly, infection with ‘*Ca.* L. asiaticus’ negatively affected the levels of all compounds involved in both arms in the early/shared steps as well as downregulated their biosynthetic genes [56]. Collectively, these findings suggest that the blotchy mottle symptom of HLB is due to the degradation of chlorophylls, xanthophylls, and carotenes rather than chlorophylls only (Figure 3).

Additionally, the accumulation of ABA might contribute to HLB symptom development. Our previous study showed that infection with ‘*Ca.* L. asiaticus’ induced the accumulation of zeaxanthin and ABA and upregulated their biosynthetic genes [56]. Both compounds are involved in late/specific steps of the carotenoid biosynthesis pathway. It has been reported previously that the *β*-carotene pool is tightly regulated in photosynthetic tissues [69] and it is slightly converted to zeaxanthin via the activity of *β*-carotene hydroxylases (aka carotenoid hydroxylase *β*-ring (*CHYb*)) enzyme [70,71,72]. It is worth noting that *CHYb* was upregulated upon ‘*Ca.* L. asiaticus’ infection [56], which suggested that *β*-carotene is quickly metabolized to zeaxanthin. Subsequently, zeaxanthin is catalyzed to violaxanthin then to neoxanthin via the activity of zeaxanthin epoxidase (*ZEP*) and neoxanthin synthase (*NSY*), respectively [70,71,72]. Interestingly, both genes (*ZEP* and *NSY*) and other ABA biosynthetic genes, including capsanthin/capsorubin synthase (*CCS*), 9-*cis*-epoxycarotenoid dioxygenase 3 (*NCED*), short-chain alcohol dehydrogenase (*ABA2*), and abscisic aldehyde oxidase (*AAO3*), were upregulated upon ‘*Ca.* L. asiaticus’ infection, which leads to the accumulation of ABA [56,58,73]. High ABA levels induced leaf yellowing in cut Stock (*Matthiola incana*) flowers [74] and rice (*Oryza sativa*) plants [75]. Taken together, these findings propose that ABA may play a role in HLB symptom development (Figure 3); however, the molecular mechanism behind this role is still unclear and requires more investigation.

Besides, polyamines might be involved in botchy mottle symptom via the production of H_2_O_2_. Briefly, diamine oxidase (*DAO*) and polyamine oxidase (*PAO*) catalyzes the oxidation of putrescine and spermidine, respectively, to produce GABA, resulting in the accumulation of H_2_O_2_ within the apoplast [76,77]. The accumulation of H_2_O_2_ in citrus plants after infection with ‘*Ca.* L. asiaticus’ has been previously reported [78]. In our previous study, GABA was accumulated and both D-amino acid oxidase PA4548 (aka diamine oxidase *CsDAO*) and polyamine oxidase 1 (*CsPAO*) were expressed at higher levels in ‘*Ca.* L. asiaticus’-infected plants [49]. Thus, we hypothesize that the induction of PAs and their catabolic genes (*CsDAO* and *CsPAO*) are correlated with the accumulation of GABA and H_2_O_2_, which work together to create an incompatible interaction between the host and the pathogen. Nevertheless, the alteration in *DAO* and *PAO* and the accumulation of H_2_O_2_ have to occur in the appropriate location of the plant to be effective in the creation of incompatible interactions [77]. Interestingly, ‘*Ca.* L. asiaticus’ could survive the toxic effects of accumulated H_2_O_2_ using its own peroxidase [78,79]. The detoxification system of citrus plants, however, might not be sufficient to reduce the high H_2_O_2_ levels, which may eventually become toxic to the leaf tissue and cause the characteristic blotchy mottle symptom that appears after ‘*Ca.* L. asiaticus’ infection [78].

### 3.2. The Role of Citrus Metabolites in the Development of Leathery Leaf Symptom

Leathery leaves are a distinctive symptom of HLB, particularly at the later phases of the disease. For instance, ‘*Ca.* L. asiaticus’-infected trees of sour orange (*C. aurantium*) and key lime (*C. aurantiifolia*) showed thicker and leathery leaves in advanced stages of the disease compared with noninfected plants [12,40]. However, the physiological and molecular mechanisms behind how this symptom developed are poorly understood. Herein, we suggest that the accumulation of starch and auxins might be involved in the development of leathery leaf symptom (Figure 3). Our earlier study showed that infection with ‘*Ca.* L. asiaticus’ induced starch accumulation in the infected leaves [56]. Additionally, a massive accumulation of starch grains was observed in the photosynthetic cells, phloem elements, vascular parenchyma, and all other parenchyma cells of ‘*Ca.* L. asiaticus’-infected leaves and petioles [40,80,81]. Together, these findings suggest that starch accumulation partially explains the development of leathery leaf symptom. Likewise, auxin accumulation might be involved in the development of leathery leaf symptom. Previously, we showed that infection with ‘*Ca.* L. asiaticus’ and the infestation with *D. citri* increased the levels of three detected auxins, including indole-3-acetic acid (IAA), indole-3-propionic acid (IPA), and indole-3-butyric acid (IBA) [58]. Moreover, exogenous application of cotton (*Gossypium hirsutum*) plants with α-naphthalene acetic acid (NAA; a synthetic auxin-like plant growth regulator) triggered the development of thicker and more leathery leaves [82]. Collectively, these findings support our suggestion that auxin accumulation is involved in the development of leathery leaf symptom of HLB (Figure 3).

### 3.3. The Role of Citrus Metabolites in the Reduction of New Flushes

At severe infection stages, ‘*Ca.* L. asiaticus’ infection diminishes the development of new flushes on the HLB-infected trees or it forms shoots with compressed internodes and small upright leaves, showing a symptom commonly known as “rabbit-ears” [83,84]. We believe that phytohormones (Figure 2) are intimately involved in the reduction of the frequency of vegetative flushes and leaf size (Figure 3), particularly the auxin/cytokinin ratio. Taking together the facts, (I) activation of dormant buds is a complex process and extremely finely tuned [85]; (II) several environmental factors and endogenous signals regulate the development of vegetative flushes and shoot system architecture, particularly auxins and cytokinins [85,86,87,88]; (III) auxins are synthesized in the apical meristems and then translocated basipetally (downward through the phloem) and suppress axillary bud outgrowth [85,86]; (IV) cytokinins are synthesized in the root tip meristems, travel acropetally (upward through the xylem), and promote/stimulate axillary bud outgrowth [85,86]; (V) both auxins and cytokinins interact antagonistically and are affected by relative auxin/cytokinin ratio [85,86,87,88]; (VI) our previous study demonstrated that infection with ‘*Ca.* L. asiaticus’ increased the endogenous levels of three auxins (IAA, IPA, and IBA) but did not alter the cytokinin (*trans*-zeatin and *trans*-zeatin riboside) contents, which distorted the auxin/cytokinin ratio [58]. Collectively, we propose that the auxin/cytokinin ratio regulates the initiation and development of vegetative buds. In *Ca*. L. asiaticus’-infected trees, the higher auxin content might prevent the outgrowth of vegetative buds and decrease the frequency of new flushes. We believe that the inhibitory activity of auxins is mediated by a second signal such as cytokinins, ABA, and bud-specific proteins (Figure 3).

Additionally, the elevated ABA content following ‘*Ca.* L. asiaticus’ infection [58] might stimulate bud dormancy and reduce the development of new flushes. It is well-known that ABA and its biosynthetic genes (*NCED*, *ABA2*, and *AAO3*) are associated with the dormancy of grapevine (*Vitis* sp.) [89], peach (*Prunus persica*) [90], leafy spurge (*Euphorbia esula*) [91], and pear (*Pyrus pyrifolia*) [92]. Moreover, the exogenous application of ABA inhibited the release of grapevine buds from the dormancy stage [93]. Collectively, these findings reinforce our suggestion that ‘*Ca.* L. asiaticus’-induced ABA is involved in the reduction of new flushes of infected trees via the regulation of bud dormancy (Figure 3).

### 3.4. The Role of Citrus Metabolites in the Development of Lopsided Fruits

Lopsided fruit (also known as misshapen fruit) is one of the most iconic symptoms of HLB. Generally, most of the recent citrus fruit-related research has focused on the juice quality and physical fruit characteristics [22,40,94,95,96,97]; however, studies on the molecular and physiological effects of HLB on the growth and development of citrus fruits are very limited. We believe that the misshapen fruit symptom is a result of complex molecular, metabolic, and anatomical changes driven by ‘*Ca.* L. asiaticus’ bacterium and/or its vector, *D. citri*, which is mainly controlled by phytohormones.

Development of the misshapen fruit symptom was previously explained based on analysis of the spatial distribution of indole-3-acetic acid (IAA) and abscisic acid (ABA) in HLB-symptomatic fruit [59], abnormal growth, and cell enlargement in the subepidermal layers (hypodermis) [59], differential expression of phytohormone-related genes [46], and differential transcription of GH3-like proteins involved in auxin synthesis [60,98]. Interestingly, both IAA and ABA were asymmetrically distributed in the flavedo tissues of HLB-symptomatic fruits. Additionally, flavedo tissues removed from the large shoulder of misshapen HLB-symptomatic fruit had higher endogenous IAA levels when compared with tissue from the same fruit but on the opposite side (normal-sized area) [59]. In agreement with these findings, the upregulation of auxin- and ABA-related genes was also reported [46]. Likewise, transcripts of auxin-responsive genes, particularly GH3-like proteins (GH3.1 and GH3.4) and ABA-related genes (GRAM-domain containing protein) were higher in HLB-symptomatic fruit [60,98].

It is well-known that elevated levels of auxins, particularly IAA, are associated with cell enlargement and citrus fruit expansion [99]. For instance, larger parenchyma cells (more than 25% larger) were microscopically observed in the hypodermis (3–5 cell layers below the epidermis) of the flavedo of HLB-symptomatic fruits and could explain the development of abnormal growth and consequently lopsided fruits [59]. Interestingly, hypodermal cell area was positively correlated with endogenous content of IAA but negatively correlated with the number of cells per unit area [59]. Collectively, these findings suggest that IAA plays a key role in cell enlargement and expansion in HLB-symptomatic fruits; however, the overall fruit size remains smaller due to the lower number of cells per unit area. Other phytohormone-related pathways such as involving ABA, gibberellins, cytokinins, jasmonates, and ethylene might be implicated in the alteration of hypodermal cell size and number in HLB-symptomatic fruit as their related genes were differentially expressed in HLB-infected fruit tissues from *C. sinensis* [46]. However, further investigations are required to quantify the endogenous levels of these phytohormones in HLB-infected fruits and to explore their roles in fruit symptom development.

### 3.5. The Role of Citrus Metabolites in HLB-Induced Preharvest Fruit Drop

Citrus species usually overproduce flowers beyond that needed for a full crop during the blooming season [100]. However, heavy flowering does not necessarily guarantee a subsequent economic crop of fruits. Poor fruit set either by severe post-bloom drop and/or preharvest drop could cause significant losses in yield. Fruit drop is a natural phenomenon that occurs in most, if not all, fruiting trees such as citrus. Citrus trees tend to naturally shed some fruitlets and immature fruit after flowering during three periods (post-bloom, June drop, and preharvest fruit drop). Herein, we will focus only on the preharvest fruit drop of mature fruit that occurs at three to four months before commercial harvest.

**Figure 4:**
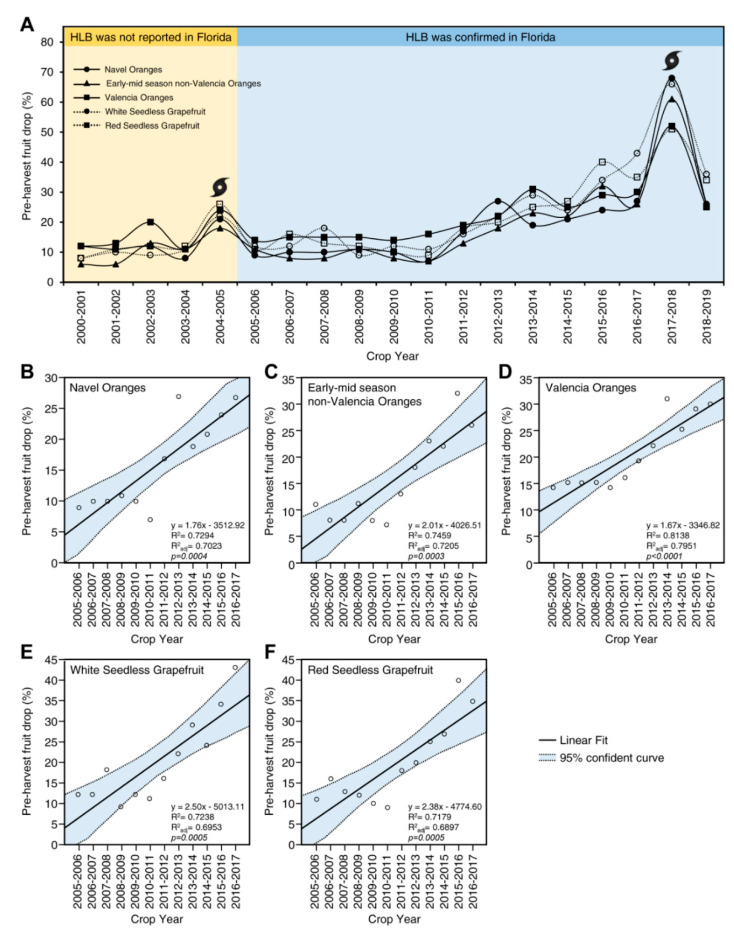
Preharvest fruit drop (%) from 2000 to 2019. (**A**) The fruit drop profile of five citrus groups including navel oranges, early-mid-season non-Valencia oranges, Valencia oranges, white, and red seedless grapefruits. The graph is based on the available information of citrus production reports in Florida from the USDA’s National Agricultural Statistics Service (NASS, 2019) available at https://www.nass.usda.gov/StatisticsbyState/Florida/Publications/Citrus/Citrus_Forecast/history.php. The dramatic increase in fruit drop during the 2004–2005 season was mainly due to the 2004 Atlantic hurricane season (Hurricane Charley, Hurricane Frances, Hurricane Ivan, and Hurricane Jeanne), whereas the high fruit drop during 2017–2018 season was due to the high winds of Hurricane Irma in September 2017. (**B**–**F**) Simple linear regression between crop season and preharvest fruit drop (%) of five citrus groups including navel oranges, early-mid-season non-Valencia oranges, Valencia oranges, white seedless grapefruits, and red seedless grapefruits, respectively. Dots present data on citrus production in Florida from the USDA’s NASS. The fitted regression line is presented as a solid line. The 95% confidence curve for the estimated linear regression is light-blue-shaded and edged by dotted lines. Regression equation, R^2^, R^2^_adj_, and *p*-values were also obtained and are presented in the lower right corner of the graph.

In citrus, HLB increases the preharvest mature fruit drop (up to three months before commercial harvest), which results in a significant reduction in yield (Figure 4) and overall grove productivity. Data presented in Figure 4A shows the preharvest fruit drop (%) over almost 20 years (from 2000 to 2019) according to Citrus Production Forecast reports in Florida from the National Agricultural Statistics Service, US Department of Agriculture [101]. In general, the percentages of preharvest fruit drop have increased gradually since HLB was first reported in Florida in 2005, punctuated by the dramatic increases in fruit drop in the 2004–2005 season due to the 2004 Atlantic hurricane season (Hurricane Charley, Hurricane Frances, Hurricane Ivan, and Hurricane Jeanne), and the 2017–2018 season, which was mainly due to the high winds of Hurricane Irma in September 2017 (Figure 4A).

The estimated preharvest fruit drop rate was increased up to 30% in navel oranges (Figure 4B), early-mid-season non-Valencia oranges (Figure 4C), and Valencia oranges (Figure 4D), and up to 40% in white and red seedless grapefruits (Figure 4E,F, respectively). The simple linear regression showed that the preharvest fruit drop rate (%) was positively correlated with the crop year (time), with the coefficient of determination higher than 70% (R^2^ > 0.7) (Figure 4). This dramatic increase in mature fruit drop is a consequence of the widespread invasiveness of ‘*Ca.* L. asiaticus’ and the epidemic of HLB in Florida, the inaccessibility of curative treatment, and the absence of HLB-resistant varieties [12,102,103].

**Figure 5:**
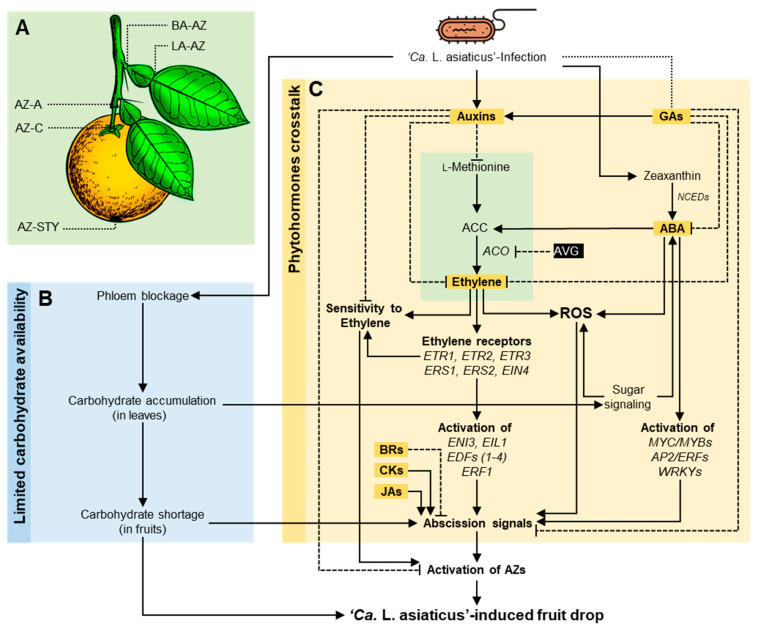
Hypothetical model of the potential roles of citrus phytohormones and carbohydrate availability in the HLB-induced preharvest fruit drop. (**A**) Citrus leaf and fruit abscission zones (AZs). There are five well-defined AZs in citrus. The leaf AZs are located between the shoot and the petiole (BA–AZ; also known as Branch–AZ or Basal–AZ) and between the petiole and the blade (LA–AZ; also known as Laminar–AZ), whereas the fruit AZs are located between the shoot and the peduncle (AZ–A), between the calyx and the fruit itself (AZ–C), and between the fruit and the style (AZ–STY). (**B**,**C**) Two hypothetical models that discuss the potential role of carbohydrate availability and phytohormone imbalance, respectively, in HLB-induced preharvest fruit drop. Briefly, the first hypothesis is based on that ‘*Ca*. L. asiaticus’ infection induces collapse and proliferation of phloem cells and plugging of sieve pores in citrus leaves causing phloem blockage, which could block the carbohydrate flow in the phloem, causing limited carbohydrate availability to the citrus fruits. The second hypothesis was built on the phytohormonal imbalance at the AZs, which might play a key role in the regulation of cell separation processes and, eventually, fruit drop. Several phytohormone groups might be implicated in abscission processes, including auxin and indole derivatives, ethylene and its precursors, abscisic acid (ABA), gibberellins (GAs), cytokinins (CKs), brassinolide (BRs), and jasmonates and their methyl ester (JAs).

We believe that HLB-induced fruit drop might be mainly due to the alteration in metabolic homeostasis at the abscission zone (AZ). In citrus, there are five well-defined fruit and leaf abscission zones (Figure 5A). The leaf abscission zones are located between the shoot and the petiole (BA-AZ) and between the petiole and the blade (LA-AZ), whereas the fruit abscission zones are located between the shoot and the peduncle (AZ-A), between the calyx and the fruit itself (AZ-C), and between the fruit and the style (AZ-STY) (Figure 5A). Until now, the physiological and molecular mechanisms behind the HLB-associated fruit drop have been poorly understood and few hypotheses discuss the potential role of both primary and secondary metabolites in HLB-associated fruit drop.

The first hypothesis is based on the limited carbohydrate availability to the citrus fruits (Figure 5B) due to the ‘*Ca.* L. asiaticus’ infection. It has been reported previously that ‘*Ca.* L. asiaticus’ infection induces collapse and proliferation of phloem cells and plugging of sieve pores in citrus leaves, causing phloem blockage [80,104,105,106], which could block carbohydrate flow in the phloem. For instance, sucrose was accumulated in HLB-symptomatic leaves [104] but decreased in HLB-infected fruits [42,94,96], which suggests that sugar transportation in the phloem is completely or partially blocked. Additionally, our previous study showed that starch was also accumulated in ‘*Ca.* L. asiaticus’-infected leaves [56]. On the other hand, sucrose content was lower in the peels of HLB-infected mature fruits compared with healthy fruits [59]. Interestingly, carbohydrate shortage is the predominant cause of the June drop in citrus. Collectively, these findings suggest that ‘*Ca.* L. asiaticus’ infection might block carbohydrate flow in the phloem, causing limited carbohydrate availability to the fruit, which increases the preharvest fruit drop in a similar way to June drop.

Another hypothesis was built on the phytohormonal imbalance (Figure 5C) at the abscission zone (AZ), which might play a key role in the regulation of cell separation processes and, eventually, fruit drop [99,100,107,108,109]. The role of endogenous phytohormones in the activation of AZ has previously been intensively investigated. Several phytohormone groups might be implicated in abscission processes (Figure 5C). These groups including auxin and indole derivatives [110,111], ethylene and its precursors [112,113,114,115], abscisic acid [116], gibberellins [117,118], cytokinins [119], brassinolide [120], and methyl-jasmonate [121]. Phytohormones regulate the abscission process via a complex response network where they work as accelerating or inhibiting signals [109]. The role of phytohormones mainly relies on their endogenous levels in different tissues, concentrations, affinities of their receptors and their homeostasis, transport, and/or interactions with each other (crosstalk). Generally, ethylene [113], cytokinins [119], abscisic acid [108], and methyl-jasmonate [121] act as abscission-accelerating signals (Figure 5C), whereas auxins [111], gibberellins [118], and brassinolide [120] act as abscission-inhibiting signals [109].

Since phytohormones do not function separately and are involved in whole-plant biology, it is important to discuss the crosstalk between phytohormone groups during fruit abscission. Briefly, auxins, particularly IAA, are produced by young leaves and are translocated down to AZ, where they act antagonistically in AZ activation via the reduction of its sensitivity to ethylene [122]. Interestingly, previous studies showed that auxins, particularly indole-3-acetic acid (IAA), were accumulated in HLB-infected leaves [58] and in the flavedo from the misshapen section of HLB-symptomatic fruits [59] of ‘Valencia’ sweet orange. Furthermore, the post-bloom fruit drop of citrus caused by the fungal pathogen *Colletotrichum acutatum* might be due to alteration of the balance between auxin and its related indole derivatives produced by the fungus [110]. On the other hand, the exogenous treatment with gibberellins reduced fruitlet drop in citrus via the acceleration of IAA metabolism [123]. However, gibberellin deficiency was associated with higher ABA levels and increased the ethylene release and caused ovary abscission [100,124].

Although it is well-known that ABA does not promote abscission by itself when applied to the aerial parts of the plant, it stimulates ethylene synthesis and promotes abscission [108]. In other words, previous ABA accumulation is required for ethylene-induced abscission [125]. ABA regulates the endogenous levels of 1-aminocyclopropane-1-carboxylic acid (ACC; a key precursor of ethylene), leading to the accumulation of ethylene and, thereafter, abscission [99,126,127]. It has been previously shown that ABA promotes ACC biosynthesis via the induction of ACC synthase activity [112]. Subsequently, ACC is transported upward from the roots to aerial parts of the plant through xylem flow, where it is metabolized to ethylene and promotes abscission [112]. Our previous studies showed that ABA [58] and its precursor ‘zeaxanthin’ [56] were accumulated in ‘*Ca.* L. asiaticus’-infected leaves compared with healthy leaves. Consequently, we suggest that ABA accumulation due to the ‘*Ca.* L. asiaticus’ infection might trigger ethylene production, causing abscission and fruit drop.

Many genes regulating the activation of abscission involve ABA and/or ethylene also activate a part of the phytohormone biosynthesis and signaling pathways [109] (Figure 5C). Briefly, ABA acts as a modulator of ACC content and promotes ethylene biosynthesis via activation of some specific ABA-signaling genes and transcription factors such as APETALA2/ethylene response factor (*AP2/ERF*), MYC/MYB, and WRKY transcription factors. Subsequently, ethylene is recognized by an ethylene receptor such as ethylene response (ETR; including ETR1, ETR2, and ETR3), ethylene response sensor (ERS; including ERS1 and ERS2), and ethylene insensitive 4 (EIN4), leading to the activation of several cascades and transcriptional regulators such as ethylene insensitive 3 (EIN3), carboxyl end of EIN2 (CEND), four endothelial differentiation-related factors (EDF1 to EDF4), ethylene insensitive-like 1(EIL1), and ethylene response factor 1 (ERF1) [109], which lead to the acceleration of the dissolution of the middle lamella and cell/organ separation.

Interestingly, several previous studies demonstrated that both carbohydrate availability and phytohormone balance participate together in a complex signal transduction system to regulate citrus fruit drop and abscission [114,118,127,128]. The carbohydrate–phytohormone interaction during fruit drop/abscission has been intensively investigated. For example, defoliation at anthesis promotes citrus fruitlet abscission via carbohydrate shortage and the accumulation of abscission-associated phytohormones [127,129]. Defoliation decreased the sugar content but increased the endogenous ABA and ACC contents (stress-sensitive signals) originating in fruitlets before their abscission [99]. Furthermore, the relationship between ethylene and carbohydrate levels might play a key role in citrus fruit drop [114]. The exogenous treatment with ACC and sucrose, separately or combined, increased ethylene production; however, the treatment with aminoethoxyvinylglycine (AVG; an inhibitor of ACC oxidase) decreased ethylene and ACC production. Likewise, branch girdling treatment induced carbohydrate accumulation but reduced ethylene production, and eventually reduced the abscission rates [114]. Branch girdling coincided with higher carbohydrate (hexose and starch) and gibberellin contents in developing ovaries and fruitlets in satsuma mandarin [118]. Taken together, we suggest that ‘*Ca.* L. asiaticus’ might control the fruit drop in citrus through the regulation of carbohydrate availability and phytohormone balance in a complex signal transduction system. However, more investigations are required to explore the molecular mechanisms and signaling pathways that might be involved in citrus fruit drop induced by HLB.

### 3.6. The Role of Citrus Metabolites in HLB-Associated Root Damage

Although most studies on HLB symptom development have focused on the visually apparent foliar symptoms, very few studies have focused on root-associated symptoms [44,130]. In most cases of vector-borne phloem-restricted bacterial diseases, including HLB, the role of root infection has been underestimated and considered as a secondary symptom resulting from carbohydrate starvation caused by phloem blockage [22,80,131,132,133]. HLB is a systemic disease. It has been previously shown that ‘*Ca.* L. asiaticus’ invades all phloem-containing tissues, including leaves, stem, and roots of citrus plants [21,44,134] and other experimental hosts such as periwinkle (*Catharanthus roseus*) [135].

Upon transmission, ‘*Ca.* L. asiaticus’ quickly moves throughout the phloem sieves to the root system [130]. Due to the initial movement of ‘*Ca.* L. asiaticus’ to the roots, it might cause root dieback, root collapse, and root damage that directly affects tree health, even before the appearance of foliar symptoms [44]. In addition, the HLB-symptomatic trees (partially in one branch or in the whole canopy) usually have fewer roots, lower root dry weight, and lower root density compared to presumptively healthy and HLB-symptomless trees (Figure 1F,G). Furthermore, ‘*Ca.* L. asiaticus’ infection in citrus roots might serve as an inoculum source to new foliar flushes through vascular movement of the bacterium [44]. However, the molecular mechanisms behind the early ‘*Ca.* L. asiaticus’-associated events in the root system are poorly understood and require more investigations [44,80,136].

Recently, Johnson et al. hypothesized that ‘*Ca.* L. asiaticus’ preferentially moves from the infection site to the root system where it colonizes, multiplies, damages the root system, and subsequently spreads to the shoot system during new leaf flushes, which act as the sink tissue for phloem flow [44]. These findings were partially in agreement with another study on periwinkle (*C. roseus*) where the first yellowing symptoms appeared on the shoots below the graft insertion and then gradually appeared on most leaves of infected periwinkles [135]. These results support the hypothesis that ‘*Ca.* L. asiaticus’ diffuses from the infection site (graft insertion or *D. citri*-feeding site) to the root, and that the direction of diffusion tends to be from the top to the bottom of the plant.

Additionally, ‘*Ca.* L. asiaticus’ bacterium was detected in roots prior to leaves, suggesting that roots serve as a source/reservoir of ‘*Ca.* L. asiaticus’ inoculum for infection of the new foliar flush [44]. For example, heavily pruned ‘Hamlin/Swingle’ trees with higher ‘*Ca.* L. asiaticus’ populations in roots before pruning showed more rapid colonization of new flushes after pruning. Trees with ‘*Ca.* L. asiaticus’-free roots did not show any ‘*Ca.* L. asiaticus’ detection in the new flush (up to two months post pruning) even if they had high ‘*Ca.* L. asiaticus’ populations in leaves before pruning. Moreover, in some HLB-infected trees, the ‘*Ca.* L. asiaticus’ population in roots briefly decreased after the new flush, suggesting that the bacteria traveled to the new flush from the roots [44].

Most recently, a study was carried out using HLB-infected citron, Duncan grapefruit, and Cleopatra mandarin rootstocks to evaluate the expression of some putative effectors of ‘*Ca.* L. asiaticus’ during the interaction with citrus hosts with varying degrees of tolerance toward HLB [137]. Briefly, high mRNA abundance of ‘*CLIBASIA_0*5640’ was detected in citron roots, whereas the abundance of ‘*CLIBASIA_0*3875’ was higher in roots of Duncan grapefruit and Cleopatra mandarin plants, even at low bacterial titers [137]. These findings suggest that ‘*Ca.* L. asiaticus’ might have its own molecular mechanism that contributes to HLB-induced root damage.

Additionally, the starch content of HLB-infected roots only dropped after most of the roots were lost and the foliar symptoms had appeared on the whole canopy and no anatomical changes (plugging or collapse) were reported in the phloem at the early events of HLB symptom development [44]. Previously, it has been thought that HLB-induced root damage, which happened prior to the appearance of foliar symptoms, is a result of starch depletion or carbohydrate starvation due to phloem blockage. However, these findings demonstrated that starch depletion is not responsible for HLB-induced root damage as was commonly thought [22,44,80]. In other words, HLB-induced root damage is causal of HLB symptoms, not a result.

## 4. Association between Citrus Flushing Cycles and Population Dynamics of Both ‘*Ca.* L. Asiaticus’ and Its Vector, *D. citri*

Several previous studies have been conducted to investigate the population dynamics of ‘*Ca.* L. asiaticus’ and its relation to root growth and flushing cycles of citrus and non-citrus hosts [138,139,140,141,142] as well as within the population of its vector, *D. citri* [140,143]. In addition, numerous studies focused on the association between citrus flushing cycles and the population dynamics and demography of *D. citri* [144,145,146,147,148,149,150,151]. Generally, root growth and flushing of citrus plants are cyclic [152] and the population dynamics of ‘*Ca.* L. asiaticus’ and its vector are positively correlated with these cycles of root growth and flushing rhythm of citrus plants [138,139,140,141,142,143,144,145,146,147,148,149,150,151]. Interestingly, almost the same patterns of seasonal fluctuation were observed between flushing rhythm and the African citrus greening pathogen, ‘*Ca*. L. africanus’, and its insect vector, *T. erytreae* [153,154].

**Figure 6:**
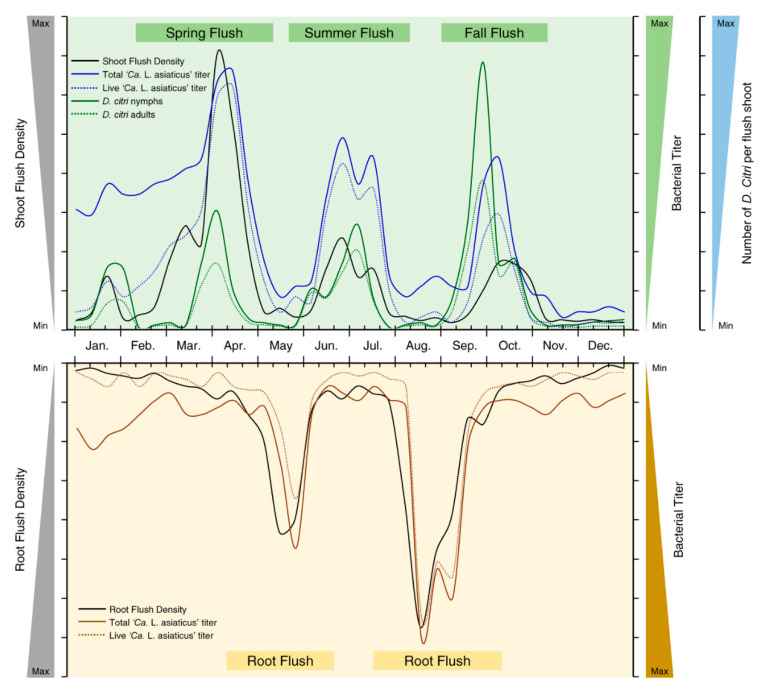
Population dynamics of ‘*Candidatus* Liberibacter asiaticus’ and its insect vector, Asian citrus psyllid, *Diaphorina citri* (Hemiptera: Liviidae), in relation to the annual flushing cycles and root growth patterns of citrus trees in Florida. The data presented in this figure were adapted from previously published data by [138,139,140,141,142,143,144,145,146,147,148,149,150,151,152].

In citriculture, the term “*flush*” describes the production of new shoot and foliar growth between bud break and shoot expansion. Seasonal flush cycles may differ based on location/region, varieties/cultivars, tree age and health, and environmental conditions [147,155]. Based on empiric observations and available published data on flush densities in Florida citrus, three major flush cycles are observed in citrus groves (Figure 6), including one main flush cycle (Spring flush) and two minor flush cycles (Summer flush and Fall flush, respectively) [142]. The Spring flush is the major flushing period that follows the cool season of the year and starts in early February to May; the Summer flush extends from early June to August, and the Fall flush begins in September/October, but very few flushes are observed between November and late January [142,147,148,149,150].

On the other hand, only two root growth cycles were observed annually. The first root growth cycle begins in late April to early June, while the second cycle extends from late July to September. Root growth cycles are largely mediated by soil temperature, soil water conditions, and/or periods of shoot elongation. Optimum root growth requires soil temperatures above 27 °C, and it might be affected negatively at soil temperatures below 22 °C [152]. Additionally, shoot flushing plays a key role in controlling the intensity of root growth. The new roots number and the root elongation rate declined corresponding to the shoot flushing cycles [152].

Interestingly, populations of both ‘*Ca.* L. asiaticus’ and *D. citri* were boosted during the flush cycles and dropped thereafter [138,140,141,142,147,148,149,150,151]. Briefly, both live and total ‘*Ca.* L. asiaticus’ populations had almost the same patterns corresponding to flush cycles of citrus plants, with significantly lower bacterial titer in December, January, and early February (Figure 6) [138,142]. Furthermore, ‘*Ca.* L. asiaticus’ was not detectable or under the limit of detection using qPCR in the new shoots of citrus plants sampled in February [141]. Likewise, the dynamics of live and total ‘*Ca.* L. asiaticus’ populations in citrus roots correspond to the seasonal root growth cycles with two peaks in May and August (Figure 6).

By contrast, the incidence of ‘*Ca.* L. asiaticus’ in the Floridian populations of *D. citri* was higher in November until January, but was relatively low (less than 10%) during the summer months (July–September), which contrasts with its incidence in citrus plants [143]. Taken together, we suggest that ‘*Ca.* L. asiaticus’ preferably spends the non-flushing periods either in citrus roots or within its insect vector through an unknown mechanism. This suggestion fits with the hypothesis that ‘*Ca.* L. asiaticus’ moves downward to the roots with phloem flow soon after infection [44,156].

Herein, we suggest a hypothetical model for the movement of ‘*Ca.* L. asiaticus’ from shoots to roots and vice versa. Briefly, during the flushing cycles, ‘*Ca.* L. asiaticus’ multiplies and colonizes new shoots. As the new shoots mature, ‘*Ca.* L. asiaticus’ moves with phloem flow from mature shoots to new root flushes, where the bacterium replicates and damages the fibrous root system [44]. Subsequently, and due to the production of the new flush cycle, ‘*Ca.* L. asiaticus’, again, moves upward to the new shoots within carbohydrate mobilization. This scenario is supported by the rapid movement of ‘*Ca.* L. asiaticus’ to the new post-pruning foliar flushes of potted trees with high ‘*Ca.* L. asiaticus’ populations in roots before pruning [44].

Similarly, the incidence of different life stages (egg, nymphs, and adults) of *D. citri* on citrus plants varies during the year. Development of *D. citri* is positively correlated with the presence of new shoots, which is essential for nymph development [147,150]. Generally, *D. citri* populations mostly recovered during flush cycles and declined thereafter [149,150]. On most hosts, three major peaks of *D. citri*, almost synchronous with the flush cycles, were recorded during the year (Figure 6); however, an additional minor peak, with lower densities of *D. citri*, was observed on lemon trees during the winter season (January) [150]. The infestation levels of *D. citri* adults dramatically increased during the fall flush cycle (September and October), whereas lower infestation levels were recorded during both spring flush (February to May) and summer flush (June to August) cycles [149,150]. Likewise, the highest densities of other developmental stages of *D. citri* (egg and nymphs) were recorded on flush shoots during the fall flush cycle in comparison with other flush cycles [149]. Recently, it has been shown that the flushing peak usually occurs about two days prior to the *D. citri* peak [151]. Collectively, we might conclude that the peaks of both ‘*Ca.* L. asiaticus’ and *D. citri* are positively correlated and nearly concurrent with the flush cycles of their hosts.

## 5. Conclusion Remarks and Future Prospects

In this review article, we comprehensively discussed the up-to-date published literature on metabolomics and transcriptomics studies to better understand the roles of citrus metabolites in the symptom development of HLB disease. Symptom development of HLB disease is a complicated process and relies on a complex metabolic network. Both primary and secondary metabolites might play a vital role in the development of HLB symptoms through the modulation of carbohydrate metabolism, phytohormone homeostasis, antioxidant pathways, or via the interaction with other metabolic pathways, particularly involving amino acids, leaf pigments, and polyamines. In citrus, the HLB symptom-associated metabolic changes may result from host cellular functions for defense reactions, or may result from the manipulation of metabolic pathways by the pathogen and its insect vector for their own benefit. We believe that the knowledge gained thus far about the roles of citrus metabolites in the development of HLB symptoms underpin our understanding of this complex pathosystem. This understanding provides valuable information for the development of innovative short- and long-term control strategies that depend on beneficial modulation of these metabolic pathways to enhance disease resistance in citrus plants. Furthermore, due to the complexity of the HLB pathosystem, phytopathologists, horticulturists, and entomologists should collaborate to find some sustainable solutions to this destructive disease.

Due to the long incubation and latent periods of ‘*Ca.* L. asiaticus’ prior to the appearance of foliar symptoms, the monitoring and diagnosis of HLB disease based on its visual symptoms could be problematic and ineffective, which greatly complicates disease control. Furthermore, visual monitoring of HLB is an expensive, labor-intensive, and time-consuming process. Moreover, due to the asymmetrically systemic distribution of ‘*Ca.* L. asiaticus’ within infected trees, the detection and confirmation of HLB-asymptomatic infections are very difficult. Taken together, this maximizes our need for developing new rapid, efficient, inexpensive, and reliable detection techniques for HLB. Nevertheless, there are still numerous unanswered questions in this area of research, that promises to be an inspiring topic for future research. Firstly, although numerous previous studies demonstrated that ‘*Ca.* L. asiaticus’ infection and/or *D. citri*-infestation induce significant changes in the profile of citrus metabolites, including amino acids, organic acids, fatty acids, phytohormones, polyamines, and other secondary metabolites, the contribution of these metabolites to the citrus response against HLB disease is poorly understood. Thus, further investigations are required for a better understanding of how citrus metabolites and their associated genes contribute to the citrus response. Secondly, because the pathogenesis mechanisms of ‘*Ca.* L. asiaticus’, including the activation of detoxification proteins upon infection, are largely unknown, we believe that further investigations are required to explore virulence factors of ‘*Ca.* L. asiaticus’, including secretion systems, putative effectors, lipopolysaccharides (LPSs), and toxin-like compounds that might be secreted into phloem elements or companion cells, causing localized cell death, necrosis, and/or phloem plugging. Last but not least, our knowledge about the molecular signaling cascades that promote HLB symptom development in citrus plants is still very limited and far from being well-defined. Therefore, deciphering the molecular signaling cascades involved in symptom development is a necessity, and could be a fresh area of research in the HLB pathosystem particularly, and in citrus physiology in general.

## 6. Summary Points

1. HLB symptom development is a complicated process, reliant on a complex metabolic network that is mainly regulated by phytohormones. This complex network consisting of at least three major molecular mechanisms including:
  - The disorder of carbohydrate metabolism is associated with source–sink disruption due to starch accumulation in leaves, but not in fruits, possibly due to the upregulation of glucose-phosphate transport (GPT).
  - Imbalance of stress-associated phytohormones of citrus plants, particularly jasmonic-salicylic acid crosstalk.
  - Activation of detoxification proteins, particularly glutathione-S-transferases (GSTs) and modulation of antioxidant pathways.
2. The blotchy mottle symptom of HLB is not due to the degradation of chlorophylls and carotenoids only, but ABA may play a positive role in HLB symptom development via the induction of leaf yellowing. In addition, polyamines, GABA, and their catabolic genes (*CsDAO* and *CsPAO*) might be involved in blotchy mottle symptom via the production of H_2_O_2_. Interestingly, ‘*Ca.* L. asiaticus’ could survive the toxic effects of accumulated H_2_O_2_ using its own peroxidase. However, the detoxification system of citrus plants might not be sufficient to reduce the high H_2_O_2_ levels, which may eventually become toxic to the leaf tissue and cause the characteristic blotchy mottle symptom.
3. The leathery leaf symptom of HLB might be due to the extensive accumulation of starch grains and/or auxins in the photosynthetic cells, phloem elements, vascular parenchyma, and all other parenchyma cells of the symptomatic leaves and petioles, causing thicker and leathery leaves.
4. Development of the misshapen fruit symptom might be due to the spatial distribution of IAA and ABA in HLB-symptomatic fruits, abnormal growth, and cell enlargement in the subepidermal layers (hypodermis), differential expression of phytohormone-related genes, and/or differential transcription of auxin-responsive genes, particularly GH3-like proteins (GH3.1 and GH3.4) and ABA-related genes (GRAM-domain containing protein).
5. HLB increases preharvest mature fruit drop, which results in a significant reduction in yield. HLB-induced fruit drop might be mainly due to the alteration in metabolic homeostasis at the abscission zone (AZ), particularly the limited carbohydrate availability to citrus fruits due to phloem blockage and phytohormonal imbalance, including of auxin and indole derivatives, ethylene and its precursors, ABA, gibberellins, cytokinins, brassinolide, and methyl-jasmonate.
6. Populations of both ‘*Ca.* L. asiaticus’ and *D. citri* were boosted during the flush cycles and dropped thereafter due to the movement downward from mature shoots to new root flushes with phloem flow, where ‘*Ca.* L. asiaticus’ preferably spends the non-flushing periods either in citrus roots or within its insect vector. Due to the production of the new flush cycle, ‘*Ca.* L. asiaticus’, again, moves upward to new shoots within carbohydrate mobilization.
